# Neglected Tropical Disease Control in the “Post-American World”

**DOI:** 10.1371/journal.pntd.0000812

**Published:** 2010-08-31

**Authors:** Peter J. Hotez

**Affiliations:** 1 Department of Microbiology, Immunology, and Tropical Medicine, George Washington University, Washington, D.C., United States of America; 2 Sabin Vaccine Institute, Washington, D.C., United States of America; Swiss Tropical and Public Health Institute, Switzerland

*We cannot expect the United States and the United Kingdom to shoulder the entire financial burden of global NTD control. The world's emerging market economies and the nations of the Gulf Cooperation Council must now step up and share this commitment.*

Writer, columnist, and *Newsweek International* Editor Fareed Zakaria has coined the term “the post-American world” to refer to a new world order that has unfolded over the last decade [1]. Briefly stated, the United States became the world's most powerful nation beginning in the 20th century, and since the fall of communism we have lived in a world in which the US is the only superpower. However, the last few years have witnessed what Zakaria calls the “rise of the rest,” referring to massive economic growth in what we ordinarily refer to as developing countries. He points out that 124 countries grew at a rate of 4% or higher in 2006 and 2007 [1]. While much of this growth can be attributed to the so-called BRIC emerging economies, i.e., Brazil, Russia, India, and China, as well as other Asian nations, the economies of at least 30 African countries also increased, and in all, poverty has been falling among 80% of the world's population, including Indonesia, Kenya, and South Africa [1]. Indeed, with the exception of about 50 truly devastated nations, there has been general global growth throughout the low- and middle-income countries of Africa, Asia, and the Americas [1].

**Figure 1:**
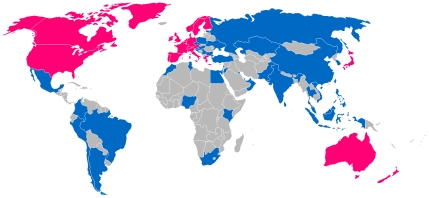
The emerging market economies. From Wikimedia Commons. Available: http://commons.wikimedia.org/wiki/File:Developed_and_Emerging_markets.png. Accessed July 21, 2010.

World Bank President Robert Zoellick has echoed similar sentiments. In an April 2010 address, he made the statement that 2009 saw the end of what was known as the “Third World” [2]. According to Mr. Zoelick, Asia's stock markets now account for a larger share of global market capitalization than those of the United States or Europe. He further states that “this change is not just about China and India” [2]. Instead, what we used to call the developing world's share of global GDP in purchasing power parity is approaching 50%, while India, Bangladesh, and sub-Saharan Africa, where most of the “bottom billion” (i.e., the world's poorest people surviving below the World Bank poverty level of US$1.25 per day) live, are each expected to grow by an average of over 6%–7% annually for at least the next five years [2]. Together, the Southeast Asian countries of Vietnam, Thailand, and Malaysia have gone from being a low-income region to becoming a powerful group of middle-income countries with important links to India and China [2]. Some of this economic growth is being fueled by the Middle East, which has become an important source of global capital. Today, the sovereign wealth fund assets of the Gulf Cooperation Council countries, meaning Kuwait, Bahrain, Saudi Arabia, Qatar, United Arab Emirates, and Oman, are estimated to be approximately US$1 trillion [2]. As a result, the influence of the G8, as well as other European countries, is evaporating in favor of a larger group of G20 countries, which includes Brazil, Mexico, China, Korea, Saudi Arabia, India, Indonesia, and South Africa (Figure 1).

Not everyone agrees with such global economic assessments, including the anthropologist Thayer Scudder, who points to evidence suggesting the overall decline of global living standards [3]. But if Zakaria and Zoellick are correct, I believe their assessments and predictions of geopolitical and economic transformations in the developing world could soon have important implications for the global control of the neglected tropical diseases (NTDs). The NTDs are the most common infections of the world's poor, especially the bottom billion [4]. Most are chronic and disabling parasitic infections such as the intestinal helminth infections, schistosomiasis, lymphatic filariasis, food-borne trematode infections, and onchocerciasis, as well as selected bacterial and viral infections such as trachoma and dengue [4], [5]. A key feature of the NTDs is their ability to exacerbate poverty by impairing child development, pregnancy outcome, and agricultural worker productivity (evidence reviewed in reference [4]). Another feature of the NTDs is their disproportionate impact on Islamic nations and rogue nuclear states, and therefore the areas of geopolitical interest to the US and Europe [6]–[9].

In response to the growing awareness of the importance of NTDs as a global health and security threat, the US government, through its Agency for International Development (USAID), and to some extent the British Department for International Development (DFID), have begun to provide up to US$100 million annually for NTD control, with plans to possibly double this amount by 2011 [10]. Additional funds for global parasite control have been provided to the government of Japan through its Hashimoto Initiative [11]. While these dollar amounts pale in comparison to the funds allocated for HIV/AIDS through the President's Emergency Plan for AIDS Relief, the truth is that these dollars will go a long way. For as little as US$0.50 annually, it is possible to provide mass drug administration to support the seven most common NTDs and those producing the highest disease burden [4], [5], [10]. Therefore, even the modest funds allocated to date may be sufficient to treat 200–400 million people annually, or roughly one-quarter of the bottom billion who urgently need treatments. In addition, ongoing support will be needed for sanitation, clean, water, and the overall strengthening of health systems [12]–[14].

Which countries should step up to treat the remainder of the world's population that urgently needs access to essential NTD medicines? Sadly, the G8 countries, other than the US, UK, and Japan, have largely ignored the NTD problem. For instance, the Italian government, which through its Ministry of Foreign Affairs held a high profile NTD workshop in Rome in 2009 [15], has so far failed to translate the conclusions of this meeting into meaningful action; similarly, the German government, whose major pharmaceutical companies, Bayer Schering and Merck KgaA, developed praziquantel in the 1970s, has failed to find a mechanism by which to establish an urgently needed public–private partnership in order fully donate this desperately needed drug for Africa [16]. In addition, the richest non-G8 European countries have also failed to support global NTD control. This failure is in stark contract to companies such as Merck & Co., GlaxoSmithKline, Johnson & Johnson, and Pfizer, which donate ivermectin, albendazole, mebednazole, and azithromycin, respectively [5]. While certainly the European governments are distracted by their own recent economic downturns, such as the recent fall of the euro and economic assistance for Greece [17], it is now clear that the disease-endemic countries must identify new funding partners for NTD development assistance.

Instead, in this new post-American world order, there is a fresh (and unprecedented) opportunity for some of the highest disease burden countries to look to what we used to call the developing world and 28 so-called emerging markets (Boxes 1 and 2). New investments from the former Third World need to happen at multiple levels. First, the BRIC countries almost certainly can afford mass drug therapeutic approaches for their own indigenous NTDs. Based on their NTD disease burden [8], [19], if the BRIC countries would take responsibility for their own control and elimination efforts, then roughly 20% of the world's burden of intestinal helminth infections, lymphatic filariasis, and trachoma could be reduced. In addition, the emerging market nations of Nigeria and Indonesia exhibit some of the highest NTD disease burdens in Africa and Southeast Asia, respectively [20], [21]. If together the BRIC countries and Nigeria and Indonesia would commit to controlling their own NTDs, it would likely double the impact of the current US commitment.

**Box 1. The Emerging Market Economies [18]**

Brazil

Chile

China

Colombia

Czech Republic

Egypt

Hungary

India

Indonesia

Malaysia

Mexico

Morocco

Nigeria

Peru

Philippines

Poland

Russia

South Africa

South Korea

Taiwan

Thailand

Turkey

**Box 2. Which Countries Should Make NTD Investments?**

- US, UK, and Japanese governments for Africa, Asia, Americas
- The remaining G8 countries and richest European countries
- BRIC countries for their indigenous NTDs
- Nigeria, Indonesia, and other emerging market economies for their indigenous NTDs
- China to support NTDs in sub-Saharan Africa
- Gulf nations to support NTDs in poor OIC countries
- Other private investments

Some of these same BRIC countries must also provide development assistance in the form of NTD control and elimination efforts for the African and Asian countries where they now heavily invest in mining, oil and gas explorations, and other activities with high economic return rates. By some estimates, Chinese investments in Africa are growing by 50% annually, and Beijing is aggressively pursuing Africa's oil, natural gas, and other reserves it requires in order to sustain growth in China [1]. According to Zakaria, at a 2006 summit held in Beijing attended by all 48 African countries, the largest number of African leaders assembled outside the continent, China promised to provide US$5 billion in loans and credits and another US$5 billion to encourage ongoing investments in the African continent [1]. Undoubtedly, some of these funds will further stimulate Africa's growing economy, but as Zakaria points out, in exchange for access to natural resources, China has entered into questionable agreements with the Mugabe regime in Zimbabwe and the Bashir regime in Sudan [1]. At a May 17, 2010 Ministerial Working Group on Scaling Up of Primary Health Systems held in Geneva and organized by Professor Jeffrey Sachs and the Earth Institute at Columbia University, I had the opportunity to meet with Dr. Chen Zhu, the Minister of Health of China, and remind him that China has an unrivaled success in NTD control, having been the first country to successfully eliminate lymphatic filariasis and make enormous progress in reducing its burden of Asian schistosomiasis [5], [21], [22]. There is now a moral and ethical imperative for China to take the lead in transferring its NTD control and elimination know-how and provide support for NTD control in sub-Saharan Africa. Given the billions of dollars China invests annually in Africa, it certainly can afford a comparatively modest investment in NTD control, one that is at least equivalent to the USAID commitment.

In addition to private philanthropic investments, the final 20% of NTD control can come from the Middle East. In a previous paper I pointed out that almost one-half of the world's NTDs occur in the member nations of the Organisation of the Islamic Conference (OIC), i.e., the world's Islamic countries [23]. In addition to Indonesia and Nigeria, the nations of Bangladesh, Chad, Mali, Niger, Somalia, Sudan, and Yemen are among the OIC countries with the highest NTD burden [23]. With the sovereign wealth funds of the Gulf Cooperation Council countries exceeding US$1 trillion [1], there is no reason why annually 0.01% of this dollar amount—US$100 million—cannot be devoted to NTD control for the poorest OIC countries.

Of course, money alone will not ensure global control and elimination of the NTDs. As new funds become available, there will be a need for appropriate financial mechanisms in order to target them for national NTD control programs; these include bilateral arrangements with health ministries in disease-endemic countries, and regional platforms and funds for the Americas, Africa, and Asia, respectively, as proposed by the Global Network for NTDs [10]. In addition, for some NTDs, there will be additional requirements to develop new drugs, vaccines, and diagnostics and then to fold these new control tools into national programs for NTD control and existing health systems [24], [25]. The major needs for research and development were outlined in a 2010 “manifesto” document for the NTDs [25], and recently the Dutch Ministry of Foreign Affairs made an important commitment to support research and development for new NTD drugs and vaccines [26]. Increasingly, there is also a global demand to strengthen health systems in parallel with support for neglected diseases [25], [27], [28]. However, there is now a desperate need for new financial support to complement current NTD control and elimination efforts by the governments of the US and UK. Today, the BRIC countries, Nigeria, Indonesia, and other emerging market economies, as well as the wealthy Gulf Cooperation Council nations in the Middle East, must agree to take on this challenge.
